# Developing a Novel Method for the Analysis of Spinal Cord–Penile Neurotransmission Mechanisms

**DOI:** 10.3390/ijms24021434

**Published:** 2023-01-11

**Authors:** Daisuke Uta, Kazuhiro Kiyohara, Yuuya Nagaoka, Yurika Kino, Takuya Fujita

**Affiliations:** 1 Department of Applied Pharmacology, Faculty of Pharmaceutical Sciences, University of Toyama, Toyama 930-0194, Japan; 2 Research Unit/Neuroscience, Sohyaku, Innovative Research Division, Mitsubishi Tanabe Pharma Corporation, Yokohama 227-0033, Japan; 3 Digital Transformation Department, Mitsubishi Tanabe Pharma Corporation, Tokyo 100-8205, Japan

**Keywords:** sexual dysfunction, sensory dysfunction, electrophysiology, in vivo extracellular recording, in vivo patch-clamp recording, spinal cord, pelvic nerve

## Abstract

Sexual dysfunction can be caused by impaired neurotransmission from the peripheral to the central nervous system. Therefore, it is important to evaluate the input of sensory information from the peripheral genital area and investigate the control mechanisms in the spinal cord to clarify the pathological basis of sensory abnormalities in the genital area. However, an in vivo evaluation system for the spinal cord–penile neurotransmission mechanism has not yet been developed. Here, urethane-anesthetized rats were used to evaluate neuronal firing induced by innocuous or nociceptive stimulation of the penis using extracellular recording or patch-clamp techniques in the lumbosacral spinal dorsal horn and electrophysiological evaluation in the peripheral pelvic nerves. As a result, innocuous and nociceptive stimuli-evoked neuronal firing was successfully recorded in the deep and superficial spinal dorsal horns, respectively. The innocuous stimuli-evoked nerve firing was also recorded in the pelvic nerve. These firings were suppressed by lidocaine. To the best of our knowledge, this is the first report of a successful quantitative evaluation of penile stimuli-evoked neuronal firing. This method is not only useful for analyzing the pathological basis of spinal cord–penile neurotransmission in sexual dysfunction but also provides a useful evaluation system in the search for new treatments.

## 1. Introduction

Peripheral nerve dysfunction is associated with several sexual dysfunctional disorders (e.g., premature ejaculation, erectile dysfunction, or sexual arousal disorder) [1,2,3,4,5]. For example, in premature ejaculation, penile sensitivity is significantly higher than that in healthy males, which may be partly attributed to afferent sensory nerve function and hyperreflexia of the ejaculatory arc [2,3]. Moreover, spinal cord and peripheral nerve neuropathy may be related to genital pain in persistent genital arousal disorder/genito-pelvic dysesthesia (PGAD/GPD) [6], and genital schistosomiasis infection may cause burning sensation and pain in the skin and dyspareunia [7,8].

Therefore, it is important to evaluate the input of sensory information from peripheral regions, such as the genital area, and the control mechanism in the spinal cord to clarify the pathophysiological basis of sensory abnormalities and pain in the genital area. In addition, inhibition of peripheral nerve firing is promising in the development of therapeutic drugs for sexual dysfunction and genital sensory disturbance [9], and an evaluation system that can analyze the neurotransmission mechanism in the genital area would be useful for pharmacological evaluation.

Genital stimuli are input to the dorsal horn of the lumbosacral spinal cord (L6–S1) via peripheral nerves [10,11,12,13]. Therefore, an in vivo evaluation system for the spinal cord–penile neurotransmission mechanism that measures the input from peripheral nerves at the genital area or at the lumbosacral spinal cord would be useful, but such an evaluation system has not yet been developed.

In this study, we developed a novel method to evaluate neuronal firing in the spinal cord and peripheral nerves following genital stimulation in rats. For the spinal cord, we developed a method to evaluate neuronal firing when the genital region was stimulated using extracellular electrophysiology in the dorsal horn of the spinal cord in the lumbosacral region or the patch-clamp method. For the peripheral nerves, we also developed a method to evaluate nerve firing in the pelvic nerve during mechanical stimulation of the genital area.

## 2. Results

### 2.1. Novel In Vivo Electrophysiological Evaluation System for Analyzing the Spinal Cord–Penile Neurotransmission Mechanism

**Figure 1:**
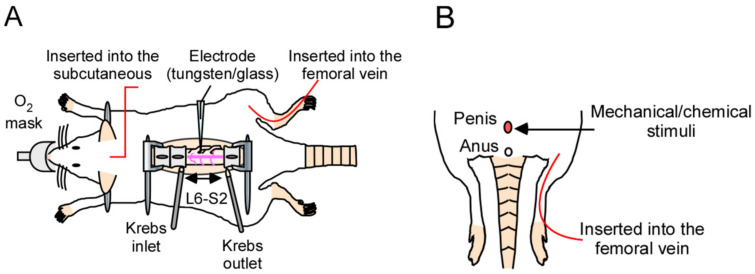
In vivo electrophysiological evaluation using extracellular or patch-clamp recordings for analyzing the spinal–penile neurotransmission mechanism in the rat spinal cord. (**A**) Schematic diagram of the in vivo electrophysiological recording method used in this study. The lumbosacral spinal cord was exposed, and the surface of the spinal cord was perfused continuously with pre-oxygenated Krebs solution (37 ± 1 °C). If drug administration is required, it can be administered intravenously or subcutaneously. The body temperature was maintained within the normal range. (**B**) Schema of the stimulation technique. The penis of the rat was directly stimulated, and the evoked firing was measured as shown in (**A**).

We attempted to evaluate evoked neuronal firing in response to penile stimulation. To record the data, we constructed a novel system for in vivo electrophysiological evaluation, including extracellular recordings and patch-clamp recordings at the dorsal horn of the spinal cord in the lumbosacral region (L6–S1), which receives input from the genital area (Figure 1A,B). This system was capable of various types of stimulation of the penis, such as tactile stimulation, noxious stimulation, and chemical stimulation. Furthermore, this system can also be used to evaluate the efficacy of drugs administered systemically by setting the route of drug administration (intravenous or subcutaneous). For detailed methodology, refer to the Materials and Methods section, and for examples of pharmacological evaluation, refer to the previous report [14].

### 2.2. In Vivo Extracellular Recordings in the Deep Spinal Dorsal Horn (Lamina III and IV) of Innocuous Stimuli-Evoked Neuronal Firing in the Genital Area

**Figure 2:**
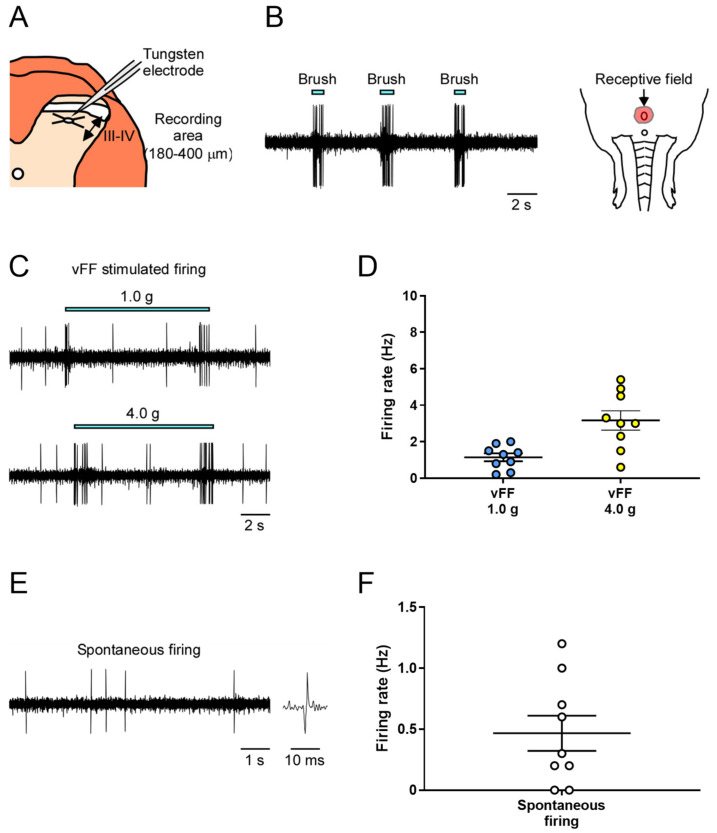
In vivo extracellular recordings in the deep spinal dorsal horn (lamina III and IV) of mechanical stimuli-evoked neuronal firing in the genital area. (**A**) Schema of the transverse slice of the lumbosacral region and a recording electrode. Neuronal firing was recorded in cells at a depth of 180–400 μm (denoted by a two-way arrow) from the surface of the spinal cord. (**B**) Representative trace of brush-evoked firing and identified receptive field. (**C**,**E**) Representative trace of vFF-evoked firing and spontaneous firing. (**D**,**F**) Quantitative evaluation of neuronal firing in response to the stimulus of vFF and spontaneous firing (n = 9 neurons from each of 9 rats). Data are presented as the mean ± S.E.M.

We conducted extracellular recordings of neuronal firing in the deep spinal dorsal horn (lamina III and IV) of the spinal cord during innocuous mechanical stimulation equivalent to tactile stimulation of the genital area (Figure 2A).

First, to identify neurons that respond to penile stimulation and their receptive fields, the penis was subjected to innocuous stimulation using a brush, and the electrode position was fixed while recording the neuronal firing evoked by the mechanical stimulation (Figure 2B).

Next, we used two different intensities of von Frey filament (vFF) (1.0 and 4.0 g) and evaluated the neuronal firing during each stimulus. We successfully recorded stimulus-evoked firing at all vFF intensities (Figure 2C). The firing frequency increased in a vFF intensity-dependent manner, allowing quantitative data analysis (Figure 2D; 1.14 ± 0.21 Hz in 1.0 g, 3.17 ± 0.53 Hz in 4.0 g). Spontaneous firing could also be assessed, but the firing frequency was not high (Figure 2E,F; 0.47 ± 0.14 Hz).

As described above, our method could evaluate stimulus intensity-dependent neuronal firing in the deep dorsal horn of the spinal cord in response to innocuous mechanical stimuli, such as touch to the penile region, and measure spontaneous firing.

### 2.3. In Vivo Extracellular Recordings in the Superficial Spinal Dorsal Horn (Lamina I and II) of Noxious Stimuli-Evoked Neuronal Firing in the Genital Area

**Figure 3:**
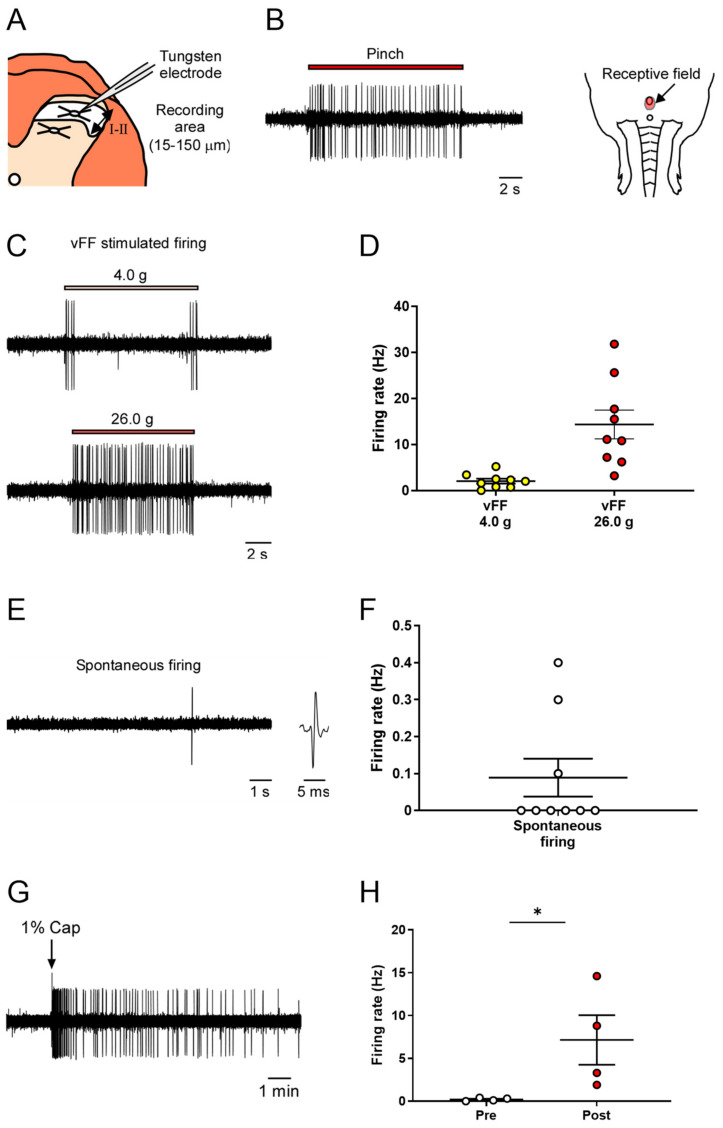
In vivo extracellular recordings in the superficial spinal dorsal horn (lamina I and II) of various stimuli-evoked neuronal firing in the genital area. (**A**) Schema of the transverse slice of the lumbosacral region and a recording electrode. Neuronal firing was recorded in cells at a depth of 15–150 μm (shown by the two-way arrow) from the surface of the spinal cord. (**B**) Representative trace of pinch-evoked firing and identified receptive field. (**C**,**E**) Representative trace of vFF-evoked firing and spontaneous firing. (**D**,**F**) Quantitative evaluation of neuronal firing in response to the stimulus intensity of vFF and spontaneous firing (n = 9 neurons from each of 9 rats). (**G**) Representative trace of capsaicin-evoked firing. (**H**) Quantitative evaluation of capsaicin-evoked neuronal firing. Data are presented as the mean ± S.E.M. * *p* < 0.05 analyzed by the Wilcoxon rank sum test (n = 4 neurons from each of four rats).

We conducted extracellular recordings of neuronal firing in the superficial spinal dorsal horn (lamina I and II) of the spinal cord during nociceptive stimulation of the genital area (Figure 3A).

As previously described in the study of the deep spinal dorsal horn, we identified neurons that respond to penile stimulation and their receptive fields by recording neuronal firing during nociceptive stimulation of the penis using forceps (Figure 3B).

Next, we used different intensities of vFF (4.0 and 26.0 g) and evaluated the neuronal firing during each stimulus. vFF stimuli-evoked firing was successfully recorded at all intensities (Figure 3C). The 4.0 g vFFs showed clear neuronal firing when the filaments were touched or the penis was released, whereas when using the 26.0 g vFF, the firing continued while the filament was pressed against the penis (Figure 3C). The firing frequency increased in a vFF intensity-dependent manner, allowing quantitative data analysis (Figure 3D; 2.10 ± 0.52 Hz in 4.0 g, 14.3 ± 3.14 Hz in 26.0 g). Similar to that in the deep layers, spontaneous firing could also be evaluated, but its frequency was not high (Figure 3E,F; 0.09 ± 0.05 Hz).

To investigate the response to chemical stimuli, capsaicin (1%) was applied to the penis as a chemical stimulant, and the evoked neuronal firing was recorded (Figure 3H; 0.20 ± 0.09 Hz in pre-administration, 7.15 ± 2.90 Hz in post-administration). Thus, our proposed method could evaluate neuronal firing in the superficial spinal dorsal horn in response to nociceptive stimuli.

### 2.4. Effect of Lidocaine on Mechanical Stimulation-Induced Neuronal Firing by vFF to the Penis in Electrophysiological Studies in the Spinal Dorsal Horn

**Figure 4:**
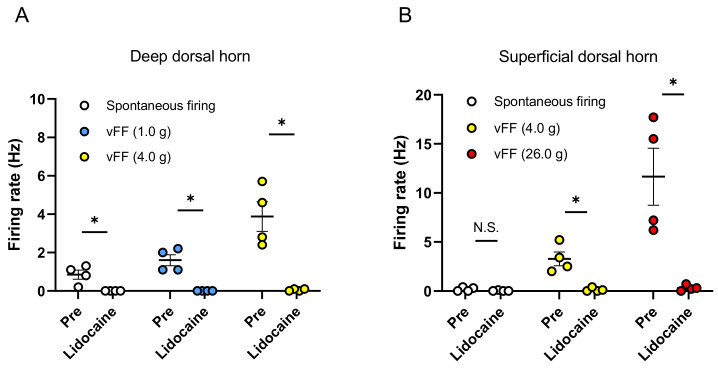
Effect of lidocaine (3 mM) on spontaneous firing and vFF-evoked firing. Summary of the inhibition effect of lidocaine in deep spinal dorsal horn (**A**) and superficial spinal dorsal horn (**B**). Data are presented as the mean ± S.E.M. * *p* < 0.05 analyzed by the Wilcoxon rank sum test (n = 4 neurons from each of 4 rats).

To confirm whether the stimulus-evoked neuronal firing recorded by this evaluation method is inhibited by the blockade of neurotransmission, we evaluated the effects of lidocaine during extracellular recording in the spinal dorsal horn. First, neuronal firing evoked by the vFF stimulation was recorded when the penis was stimulated with vFF (1.0 and 4.0 g) during extracellular recording in the deep spinal dorsal horn. These vFF-evoked neuronal firings were completely suppressed by perfusion of the spinal cord surface with lidocaine (3 mM) (Figure 4A).

Next, neuronal firing was also recorded when the penis was stimulated with vFF (4.0 and 26.0 g) during extracellular recording in the superficial spinal dorsal horn. These vFF-evoked neuronal firings were also inhibited by perfusion of the spinal cord surface with lidocaine (3 mM) (Figure 4B). After washout of lidocaine, we confirmed that the neuronal firing was recovered (no significant difference between pre- and post-washout analyzed by the Wilcoxon rank sum test).

### 2.5. In Vivo Whole-Cell Patch-Clamp Recordings in the Dorsal Horn of Neuronal Firing in the Genital Area Evoked Using Various Stimuli

**Figure 5:**
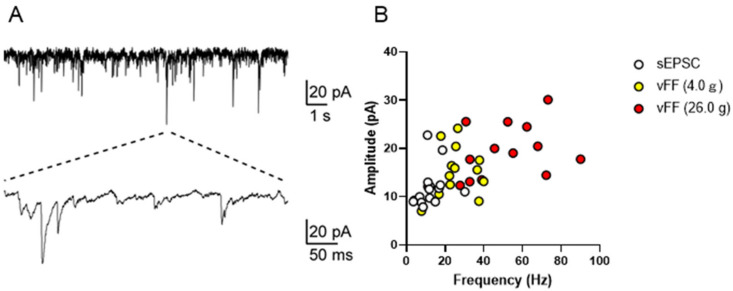
In vivo patch-clamp recording from the superficial spinal dorsal horn in the lumbosacral region. (**A**) Representative trace of sEPSCs from the superficial spinal dorsal horn. (**B**) Scatter plot showing the frequency (x-axis) and amplitude (y-axis) of spontaneous and vFF-evoked EPSCs from the superficial spinal dorsal horn (n = 14 neurons from four rats).

We investigated the possibility of using the patch-clamp method to measure neuronal firing during penile stimulation in the superficial spinal dorsal horn in the lumbosacral region. Excitatory synaptic responses were successfully recorded, and stable recordings were acquired from 14 neurons (20–120 μm depth) (representative traces are shown in Figure 5A). Specifically, the resting membrane potential of the neurons was 67.1 ± 1.2 mV (n = 14). Under conditions of a holding potential of 70 mV, neurons exhibited spontaneous excitatory postsynaptic currents (sEPSCs; a mean amplitude of 12.0 ± 1.13 pA, frequency 13.0 ± 1.73 Hz, n = 14; Figure 4A). These data were consistent with previous in vivo and in vitro reports [15,16,17]. When measurements were made under stimulation with vFF (4.0 g and 26.0 g), the mean frequency and amplitude of sEPSCs increased with the intensity of vFF, and quantitative recordings of penile stimulation-evoked synaptic responses were successfully obtained (n = 14; Figure 5B).

### 2.6. In Vivo Electrophysiological Recordings in the Pelvic Nerve of Mechanical Stimuli-Evoked Nerve Firing in the Genital Area

**Figure 6:**
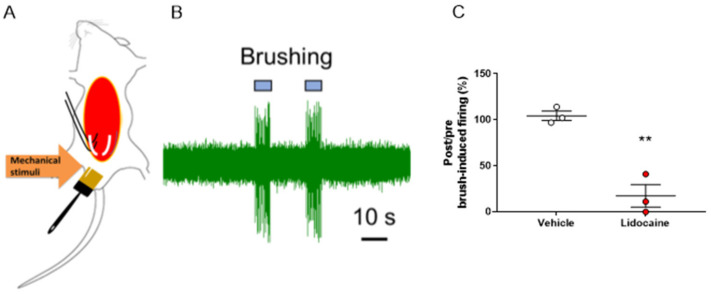
In vivo electrophysiological measurements from the pelvic nerve during mechanical stimulation of the genial area. (**A**) Schema of the in vivo electrophysiological recording. After stimulation with a brush, mechanical stimuli-evoked neuronal firing was measured. (**B**) Representative trace of mechanical stimuli-evoked firing recorded at the pelvic nerve. (**C**) Lidocaine (1.5 mM) suppressed penile mechanical stimuli-evoked firing of the pelvic nerve. The post and pre-administration ratios are shown. Data are presented as the mean ± S.E.M. ** *p* < 0.01 vs. vehicle group analyzed by Student’s *t*-test (n = three animals).

In this experiment, we attempted to evaluate the evoked nerve firing during brush-induced mechanical stimulation of the genital area. We successfully recorded nerve firing induced by mechanical stimulation of the genital area in the pelvic nerve (Figure 6A,B). Furthermore, we measured the nerve firing activity by applying lidocaine (1.5 mM) or vehicle on the exposed pelvic nerve and found that the nerve firing activity induced by brush stimulation was almost completely suppressed by lidocaine (Figure 6C).

## 3. Discussion

In this study, we established in vivo extracellular recording and patch-clamp recording methods at the spinal dorsal horn and electrophysiological recording methods at the peripheral nerve to measure neuronal activity evoked by nociceptive or innocuous stimulation of the genital area. To the best of our knowledge, this is the first methodology to quantitatively evaluate spinal cord–penile neurotransmission.

In previous electrophysiological studies of the spinal cord, pain and itch in peripheral areas, such as the limbs, have been analyzed by in vivo extracellular recording and patch-clamp recording in the lumbar spinal cord (L1–L6), which receives input from the limbs [17,18,19,20,21]. In addition, a method to analyze the characteristics of the brain-spinal cord neuronal pathway by electrophysiological studies in the lumbar spinal cord to investigate the involvement of hormones in ejaculation has been recently reported [22]. However, electrophysiological methods for detailed analysis of nerve activity during innocuous or nociceptive stimulation of the penis or other peripheral genital regions have not been established. Neuronal firing is evoked by stimulation of the genital area, such as the penis, which inputs to the spinal dorsal horn in the lumbosacral spinal cord (L6–S1) via peripheral nerves (e.g., the pelvic nerve) [10,11,12,13]. Therefore, for a detailed analysis of the neurotransmission mechanism underlying the response to penile stimulation, we attempted to apply previous techniques to the lumbosacral spinal cord region and record neuronal activity quantitatively and temporally while stimulating the penis.

Peripheral neuropathy is known to be associated with some sexual dysfunctions [1,2,3,4,5]. For example, in premature ejaculation, the nerve response to innocuous stimulation during sexual activity is impaired [2,3]. In general, innocuous stimuli are input from peripheral nerves to the deep spinal dorsal horn (lamina III–IV) via Aβ fibers [23]. In addition, neuropathies and infections cause genital pain [6,7,8], and nociceptive stimuli, such as pain, are input to the superficial layers (lamina I–II) via Aδ and C fibers [23]. Because neuronal firing activity during various types of stimulation can be analyzed in detail using extracellular recording and patch-clamp recording in the spinal cord, we used von Frey filament (vFF), which allows quantitative stimulation to record neuronal firing in the deep or superficial spinal dorsal horn. As shown in the results, it was possible to quantify the firing frequency in response to the stimulus intensity using extracellular recordings. Regarding chemical stimulation, we recorded neuronal activity during stimulation with capsaicin, a Transient Receptor Potential Vanilloid 1 (TRPV1) agonist [24,25]. Using these chemical stimulation experiments, it will be possible to analyze the contribution of various molecules to penile sensory disturbance in detail.

In this study, normal rats were used to develop a new experimental method, but it should be possible to use various pathological models for analysis. This method is also expected to be applied to mice [20] and can be used for molecular characterization using genetically modified animals. In addition, this method can be used to evaluate local drug effects in the spinal cord by adding the drug to the spinal surface perfusate. Furthermore, it is possible to evaluate the pharmacological effects of systemic administration of the drug via intravenous or subcutaneous routes of administration. We have reported a part of this methodology for characterizing histamine H_3_ receptors in response to nerve activity during penile stimulation as well as for behavioral evaluation [14].

We successfully recorded spontaneous excitatory postsynaptic currents (sEPSCs) or vFF-evoked EPSCs in the superficial spinal dorsal horn, including both extracellular and patch-clamp recordings. However, further studies on the patch-clamp recordings are needed. For example, if we can analyze not only EPSCs but also inhibitory postsynaptic currents (IPSCs) for excitatory and inhibitory signals, a detailed analysis of synaptic responses will be possible, which may contribute to a detailed investigation of the molecular mechanisms that cause sexual dysfunction.

Since in vivo electrophysiological evaluation in the spinal dorsal horn requires skilled techniques, we also investigated a brief evaluation method using peripheral pelvic nerves. This simple method is expected to be applied to screening evaluations in drug discovery research for peripheral sensory abnormalities in sexual dysfunction diseases. To investigate the findings of this method in detail, it is possible to take a multifaceted approach, such as using in vivo electrophysiological studies in the spinal dorsal horn.

## 4. Materials and Methods

### 4.1. Animals

Wistar rats (7 weeks old; Japan SLC, Inc., Hamamatsu, Japan) were used for the electrophysiological tests in the spinal dorsal horn performed at the Toyama University. Wistar–Imamichi rats (8–9 weeks old; Institute for Animal Reproduction, Ibaraki, Japan) were used for the electrophysiological tests at the pelvic nerve conducted at the Mitsubishi Tanabe Pharma Corporation (MTPC).

All rats were housed under a controlled 12/12-light cycle (lights were on from 7:00 A.M. to 7:00 P.M.), temperature was 23 ± 3 °C, and humidity was 30–70%, with access to food and water ad libitum.

All in vivo experimental procedures were performed in accordance with the “Guiding Principles for the Care and Use of Animals in the Field of Physiological Sciences” of the Physiological Society of Japan and were approved by the Institutional Animal Care and Use Committee of Research Laboratories at the University of Toyama and MTPC. All efforts were made to minimize animal stress and the number of animals used.

### 4.2. In Vivo Extracellular Recording from the Adult Rat Spinal Dorsal Horn Neurons

Recordings of the adult rat spinal dorsal horn neurons were performed using a modification of previous methods [17,21,22]. After urethane anesthesia (1.5 g/kg, intraperitoneal administration (i.p.)) of the rats, a thoracolumbar laminectomy was performed to expose the lumbar 6 (L6) to sacral 2 (S2). These rats were then placed in a stereotaxic instrument. The dura and arachnoid membranes were removed to create an access point for the electrode. The surface of the spinal cord was perfused with 95% O_2_–5% CO_2_-balanced Krebs solution (8–12 mL/min) at 37 ± 1 °C. In vivo extracellular recordings were performed from the lumbosacral region (L6–S1), which receives input from the penile region (lower abdomen). Single-unit recordings of the deep spinal dorsal horn (principally lamina III and IV) or the superficial spinal dorsal horn (principally lamina I and II) neurons were performed. Next, the spikes were selected for amplitude identification. A tungsten microelectrode (tip diameter: 25 μm, tip impedance: 9–12 MΩ) was inserted into the ipsilateral spinal cord at an angle of 20–30° (latero-medial) and recorded from neurons 180 to 400 μm below the surface (corresponding to lamina III and IV) or 15 to 150 μm below the surface (corresponding to lamina I and II). The unit signals were amplified (EX1; Dagan Corporation, Minneapolis, MN, USA), digitized (Digidata 1400A, Molecular Devices, Union City, CA, USA), and displayed online using a special software package (Clampfit version 10.2; Molecular Devices). We searched for areas on the skin where neurons responded to a light brush touch or pinching with tweezers. Mechanical stimulation was applied to the penile region for 10 s using a von Frey filament (vFF).

Capsaicin (FUJIFILM Wako Pure Chemical Corporation, Osaka, Japan) was dissolved in ethanol, and the solution was applied to the penis. Lidocaine (FUJIFILM Wako Pure Chemical Corporation) was dissolved in Krebs solution and applied while perfusing through a three-way stop-cork.

### 4.3. In Vivo Patch-Clamp Recording from the Adult Rat Spinal Dorsal Horn Neurons

Blind whole-cell voltage-clamp recordings were obtained as in previous methods [17,19,21,22,26]. The electrodes used in this study were pulled from thin-walled borosilicate glass capillaries (o.d. 1.5 mm) using a puller (p-97, Sutter Instrument, Novato, CA, USA) and filled with a solution containing potassium gluconate solution (in mM): K-gluconate 135, KCl 5, CaCl_2_ 0.5, MgCl_2_ 2, EGTA 5, HEPES 5, and ATP-Mg 5 (pH 7.2). The potassium gluconate solution was used during the recordings of spontaneous excitatory postsynaptic currents (sEPSCs) at a holding potential of −70 mV. The resistance at the tips of the patch pipettes was 6–12 MΩ. Using a micromanipulator (Model MHW-4-1, Narishige, Tokyo, Japan), the electrode was advanced at an angle of 30 from the window into the lamina I–II and formed a gigaohm seal with a cell at a depth of 20–120 μm from the contact point to the dorsal surface of the spinal cord. Series resistance was evaluated based on the response to a 5-mV hyperpolarizing step. This value was monitored during the recording session, and data were rejected if values changed by more than 15%. Signals were obtained using a patch-clamp amplifier (Axopatch 200B, Molecular Devices). The data were digitized using an analog-to-digital converter (Digidata 1400A, Molecular Devices), stored using a data acquisition program (Clampex version 10.2, Molecular Devices), and analyzed with the software package (Clampfit version 10.2, Molecular Devices). To record EPSCs, cell recordings were performed in voltage-clamp mode at holding potentials of −70 mV [17,21,22].

### 4.4. In Vivo Electrophysiological Recording from the Pelvic Nerve

All rats were anesthetized with urethane (1.6 g/kg, i.p.). After fixation in the supine position, a median incision was made in the skin and muscles from the lower abdomen to the lower diaphragm. The surrounding connective tissue was dissected to expose the pelvic nerves. Pelvic nerves were placed on a silver–silver chloride bipolar electrode connected to a bioelectric amplifier (DAM 80, World Precision Instruments, Sarasota, FL, USA) in an electric shield, and then, neuronal firing activity was recorded. Micro1401-3 (Cambridge Electronic Design, Cambridge, UK) was used as the interface. The nerve firings to be evaluated were defined as those evoked by stimulation of the genital area around the penis with a brush, and an average of two or more responses to 10 s of stimulation was used. Data were analyzed using Spike 2 software (Cambridge Electronic Design).

Lidocaine (Xylocaine solution 4%, Aspen Japan, Tokyo, Japan) was dissolved in saline, and the solution was applied directly to the pelvic nerve.

### 4.5. Statistical Analysis

Data are presented as the mean ± standard error of the mean (S.E.M.). For evaluation in the pelvic nerve, nerve firing before applying lidocaine was set to 100% and compared to change after administration. For statistical analyses, we performed a one-way analysis of variance (ANOVA), followed by a two-tailed Wilcoxon rank sum test and a Student’s *t*-test. Differences were considered significant at *p* < 0.05. All statistical analyses were performed using the SAS software (version 9.4, SAS Institute, Inc., Cary, NC, USA). In this study, *n* refers to the number of neurons (in vivo extracellular recording and patch-clamp recording) or animals (in vivo electrophysiological recording from the pelvic nerve).

## 5. Conclusions

In this study, using in vivo extracellular recording, patch-clamp recording, and electrophysiological evaluation methods in peripheral nerves, we succeeded in recording penile stimulation-induced neuronal firing and synaptic current input to the spinal dorsal horn in the lumbosacral region via peripheral nerves. This is the first report on a methodology for the quantitative evaluation of neuronal activity during penile stimulation. Using this method, it is also possible to record and analyze the frequency of neuronal firing and synaptic currents in response to various types of nociceptive and innocuous stimuli. In addition, pharmacological studies using this method can be administered in the same manner and dosage as different behavioral pharmacological studies. Therefore, this method can be a useful evaluation system not only for analyzing the pathological basis of spinal cord–penile neurotransmission mechanisms in sexual dysfunction, which have not been well studied, but also for searching for new methods of treatment as well as therapeutic drugs.

## Data Availability

The original contributions presented in the study are included in the article, and further inquiries can be directed to the corresponding author.

## References

[1] Podnar S., Vodušek D.B. (2015). Sexual dysfunction in patients with peripheral nervous system lesions. Handb. Clin. Neurol..

[2] Vignoli G.C. (1978). Premature ejaculation: New electrophysiologic approach. Urology.

[3] Xin Z.C., Chung W.S., Choi Y.D., Seong D.H., Choi Y.J., Choi H.K. (1996). Penile sensitivity in patients with primary premature ejaculation. J. Urol..

[4] Hicks C.W., Wang D., Windham B.G., Selvin E. (2021). Association of peripheral neuropathy with erectile dysfunction in US men. Am. J. Med..

[5] Azadzoi K.M., Siroky M.B. (2010). Neurologic factors in female sexual function and dysfunction. Korean J. Urol..

[6] Goldstein I., Komisaruk B.R., Pukall C.F., Kim N.N., Goldstein A.T., Goldstein S.W., Hartzell-Cushanick R., Kellogg-Spadt S., Kim C.W., Jackowich R.A. (2021). International Society for the Study of Women’s Sexual Health (ISSWSH) review of epidemiology and pathophysiology, and a consensus nomenclature and process of care for the management of persistent genital arousal disorder/genito-pelvic dysesthesia (PGAD/GPD). J. Sex. Med..

[7] Schistosomiasis. https://www.who.int/news-room/fact-sheets/detail/schistosomiasis.

[8] Engels D., Hotez P.J., Ducker C., Gyapong M., Bustinduy A.L., Secor W.E., Harrison W., Theobald S., Thomson R., Gamba V. (2020). Integration of prevention and control measures for female genital schistosomiasis, HIV and cervical cancer. Bull. World Health Organ..

[9] Althof S.E., McMahon C.G., Waldinger M.D., Serefoglu E.C., Shindel A.W., Adaikan P.G., Becher E., Dean J., Giuliano F., Hellstrom W.J. (2014). An update of the International Society of Sexual Medicine’s guidelines for the diagnosis and treatment of premature ejaculation (PE). J. Sex. Med..

[10] Taylor D.C., Korf H.W., Pierau F.K. (1982). Distribution of sensory neurons of the pudendal nerve in the dorsal root ganglia and their projection to the spinal cord. Horseradish-peroxidase studies in the rat. Cell Tissue Res..

[11] Hubscher C.H. (2006). Ascending spinal pathways from sexual organs: Effects of chronic spinal lesions. Prog. Brain Res..

[12] Carro-Juárez M., Rodríguez-Manzo G. (2008). The spinal pattern generator for ejaculation. Brain Res. Rev..

[13] Tanahashi M., Karicheti V., Thor K.B., Marson L. (2012). Characterization of bulbospongiosus muscle reflexes activated by urethral distension in male rats. Am. J. Physiol. Regul. Integr. Comp. Physiol..

[14] Kiyohara K., Uta D., Nagaoka Y., Kino Y., Nonaka H., Ninomiya-Baba M., Fujita T. (2022). Involvement of histamine H3 receptor agonism in premature ejaculation found by studies in rats. Int. J. Mol. Sci..

[15] Ohashi N., Uta D., Sasaki M., Ohashi M., Kamiya Y., Kohno T. (2017). Acetaminophen metabolite N-acylphenolamine induces analgesia via transient receptor potential vanilloid 1 receptors expressed on the primary afferent terminals of C-fibers in the spinal dorsal horn. Anesthesiology.

[16] Uta D., Hattori T., Yoshimura M. (2020). Effect of alpha 1-adrnoceptor antagonists on postsynaptic sensitivity in substantia gelatinosa neurons from lumbosacral spinal cord in rats using slice patch-clamp technique for mEPSC. Int. Neurourol. J..

[17] Uta D., Koga K., Furue H., Imoto K., Yoshimura M. (2021). L-bupivacaine inhibition of nociceptive transmission in rat peripheral and dorsal horn neurons. Anesthesiology.

[18] Furue H., Narikawa K., Kumamoto E., Yoshimura M. (1999). Responsiveness of rat substantia gelatinosa neurones to mechanical but not thermal stimuli revealed by in vivo patch-clamp recording. J. Physiol..

[19] Uta D., Kato G., Doi A., Andoh T., Kume T., Yoshimura M., Koga K. (2019). Animal models of chronic pain increase spontaneous glutamatergic transmission in adult rat spinal dorsal horn in vitro and in vivo. Biochem. Biophys. Res. Commun..

[20] Kiguchi N., Uta D., Ding H., Uchida H., Saika F., Matsuzaki S., Fukazawa Y., Abe M., Sakimura K., Ko M.C. (2020). GRP receptor and AMPA receptor cooperatively regulate itch-responsive neurons in the spinal dorsal horn. Neuropharmacology.

[21] Uta D., Tsuboshima K., Nishijo H., Mizumura K., Taguchi T. (2021). Neuronal sensitization and synaptic facilitation in the superficial dorsal horn of a rat reserpine-induced pain model. Neuroscience.

[22] Uta D., Oti T., Sakamoto T., Sakamoto H. (2021). In vivo electrophysiology of peptidergic neurons in deep layers of the lumbar spinal cord after optogenetic stimulation of hypothalamic paraventricular oxytocin neurons in rats. Int. J. Mol. Sci..

[23] Todd A.J. (2010). Neuronal circuitry for pain processing in the dorsal horn. Nat. Rev. Neurosci..

[24] Caterina M.J., Schumacher M.A., Tominaga M., Rosen T.A., Levine J.D., Julius D. (1997). The capsaicin receptor: A heat-activated ion channel in the pain pathway. Nature.

[25] Uta D., Furue H., Pickering A.E., Rashid M.H., Mizuguchi-Takase H., Katafuchi T., Imoto K., Yoshimura M. (2010). TRPA1-expressing primary afferents synapse with a morphologically identified subclass of substantia gelatinosa neurons in the adult rat spinal cord. Eur. J. Neurosci..

[26] Uta D., Yoshimura M., Koga K. (2019). Chronic pain models amplify transient receptor potential vanilloid 1 (TRPV1) receptor responses in adult rat spinal dorsal horn. Neuropharmacology.

